# Hospital and Institutional News

**Published:** 1917-08-25

**Authors:** 


### HOSPITAL AND INSTITUTIONAL NEWS.

### THE LIMITS OF INSTITUTIONAL INTERFERENCE.

The State Children's Association, which exists to maintain the principle that the home-bred child is better than the child brought up in an institution, has received a great opportunity in the visit of Judge Neil to this country and in his exposition of the now well-known Mothers' Pension Scheme. To invite him has been the most useful thing which the Association has done, a thing of more practical value than all the Association's other efforts put together. It is one thing to argue that the home-bred child was better brought up and more economical than the child removed from its home and thrust into an expensive institution. It is another to keep that home together. When the home is unsuitable for children, only two alternatives exist. The one, the usual tinkering, expensive, and sometimes cruel step of breaking it up and removing the children from their parents; the other, the sensible, humane, economical way of giving the mother money, so that she shall at least be able to keep the home decently together if she can. The latter is Judge Neil's way, now we presume, embraced, professed, adopted and placed in the front of its activities by the State Children's Association. Its title hardly indicates its bias. Institutions, we are at last beginning to learn, are evils, necessary often, but not nearly so necessary as we have supposed. It is better to supply those who now have recourse to them with sufficient money to provide their benefits for themselves. Hospitals and schools are obvious exceptions. Reformatories and industrial schools are not. Poverty is the root of many of those evils which institutions are founded to check. The Mothers' Pension Scheme deals with the poverty. The C.O.S., for instance, is noted because it does not, it merely co-ordinates the "agencies" which prey like bacilli over the sore places of our social system, and which, in the guise of remedies, are really themselves part of the disease.

### THE HEALTH OF THE SALONICA ARMY.

Since the British troops first landed at Salonica the question of the health of the men has been of the greatest importance, and the admissions to the hospitals have consisted almost entirely of sick, the proportion of wounded being very small. The main diseases met with there are malaria, dysentery, and enteric fever. The rate of sickness was at one time very high, and it is therefore very satisfactory to learn from a War Office report that the health of the troops shows a marked improvement. In proportion to the strength of the Force the admissions to hospital during July 1917 show a reduction of one-third compared with July 1916, and deaths from all diseases a reduction of nearly two-thirds. The cases of malaria are slightly fewer in number than last year, in spite of the fact that many of the cases are occurring in men who contracted the disease last year. Dysentery and diarrhœa are appreciably less prevalent. It is of interest to note that the admission rate for fevers other than malaria shows a reduction of nearly four-fifths—that is to say, there are only about one-fifth of the cases admitted during the same period last year.

### THE TROOPS AT ADEN.

It was stated in the House of Commons that there had been no report of excessive sickness amongst the troops at Aden, and it is now officially announced that although returns of the health of the troops at Aden received up to July 1917 show rather high rates of admission, yet the figures are an improvement on those of the corresponding six months in 1916. Happily, the cases admitted into hospital have, on the whole, been slight, mild attacks of malaria and "sand-fly" fever predominating. Fortunately, enteric and dysentery have been almost entirely absent. The actual figures for the first six months of this year are 60 per cent. for British troops and 30 per cent. for Indian troops. When it is borne in mind that during this time the troops have been constantly engaged in minor operations against the Turks, and the heat has been very great, the figures may be considered not very unsatisfactory.

### A SCHOOL FOR HOSPITAL MANAGERS.

A writer in the *Daily Chronicle* has given some account of the saving effected in the administration of the military hospitals, in consequence of an economy campaign started by Sir Alfred Keogh a year ago. The writer, after describing the work of Dr. Napier Burnett and Mr. Orde in this connection, which he says has saved the country two and a-half millions in a year, goes on to suggest that a similar investigation would lead to similar economies in "the tremendous sums of money demanded by the civil hospitals." "Indeed," he adds, "Dr. Burnett is disposed to think that we shall probably set up some school for hospital administration which will revolutionise the present system." He concludes by imagining that "some staggering figures will be revealed when the accounts of our civil hospitals are submitted to an expert scrutiny." The familiar loud cry of the amateur is here revealed. Has he never heard of King Edward's Hospital Fund and its control over expenditure, and of the Uniform System of Accounts? Dr. Burnett probably knows better. But it would be interesting to know what "school for hospital administration" Dr. Burnett really has in mind.

### WARNINGS OF AIR-RAIDS.

This question has proved somewhat complicated, and we can sympathise with the Home Secretary in not wishing to make the warnings so numerous as to interfere with the promptitude desired. There is, therefore, no new departure in his recent announcement that preliminary warnings of air-raids are distributed by telephone to those hospitals which have to prepare to receive casualties. While these warnings are necessarily to continue, it is not proposed to include in them those hospitals which have, or are likely to have, no preparations to make. To warn all would interfere with the rapid working of the arrangements, and it is difficult to see what would be gained by doing so.

### THE EXTENSION OF NEWCASTLE INFIRMARY.

The Newcastle Infirmary extension scheme, involving the transfer to the hospital of 15 acres, has advanced an important stage now that a Committee of the House of Commons has heard evidence in the private Bill of the Lord Mayor and the citizens, empowering them to convey to the infirmary's trustees a portion of the Castle Leazes. Mr. Whitley presided, and after Lieutenant Cripps, for the promoters, Mr. W. W. Gibson, for the infirmary, and representatives of the City Council and the Freemen had given evidence, the Chairman stated that the Committee was satisfied. Although the land required amounts to 15 acres, this is only a very small portion of the estate belonging to the City, which extends to 1,000 acres. Certain footpaths across the land will have to be closed, but another path has been arranged. We need not repeat the details which The Hospital has already published in connection with this conveyance, nor of the friendly spirit in which all parties to it have approached the question. It is a scheme with immediate and future possibilities. Mr. Gibson declared that the money was probably available for the immediate undertaking, but that for the large permanent block of buildings eventually to be constructed an appeal would have to be made.

### ONE HUNDRED THOUSAND INTERESTED HOSPITAL WORKERS.

The half-yearly meeting of the Leicester Saturday Hospital Society, held at the Leicester Royal Infirmary, was attended by about 200 delegates. The committee's report showed that 320 soldiers had been received into the Desford Hall Home during the six months. To the Society's remaining Home 141 male and 186 female members of the Society had been admitted, and 64 members had been sent to other homes, a total of 391 members thus receiving benefit. The expenditure of the Society for the six months was £3,109, as compared with £2,599 for the corresponding period of 1916. The increase was largely due to the cost of maintenance of the Desford Home, where the military patients had cost £1,829, as compared with £1,430 in the same period of last year. The Society's reserve funds were invested in a holding of £6,000 of the War Loan. Mr. Gimson, the vice-president, who was in the chair, in moving the adoption of the report, made the interesting announcement that he computed that there were approximately 100,000 persons contributing to the Society, which was another way of saying that that large number of workers were interested in the work of the Leicester Royal Infirmary and the Saturday Hospital Society. All bodies of workpeople contributing £5 5s. a year for two or more years were entitled to elect representatives to attend the quarterly meetings of the governors of the infirmary, and an interesting discussion took place as to the best means of nominating representatives, in order that the governors of the infirmary might realise the very deep interest which the contributors to the Society took in the work of the infirmary. Mr. Fielding Johnson, the chairman of the infirmary board, indicated the readiness of the governors to consider any representations made by the Society, to which they were so greatly indebted for large annual contributions for the maintenance of the institution.

### A NEW SHELL-SHOCK HOSPITAL.

The new hospital at Golders Green for the treatment of shell-shock cases having started upon its useful career, another hospital devoted to similar work will shortly be available for the use of non-commissioned officers and men, and will be situated near Manchester. This additional accommodation is the outcome of the generosity of Mr. John Leigh, of Beech Lawn, Altrincham, who, early in the year, gave the John Leigh Memorial Hospital at Altrincham to the British Red Cross Society. He now presents to the Government a large house at Brooklands, near Manchester, for the above-mentioned purpose. The house, which was the residence of Mr. Leigh's father, is fully equipped, and is to be the property of the nation for five years. The necessary alterations to the building and the equipment will involve an expenditure of several thousand pounds. Accommodation is to be provided for about 100 beds, and there will be large recreation-rooms, a large dining-hall, retiring apartments, and private quarters for the re^ident medical and nursing staffs. Mr. and Mrs. Leigh have undertaken to take an active share in management, and at the end of the five years Mr. Leigh will be prepared to consider to what other useful purpose the hospital may be devoted.

### HOSPITAL LEGACIES.

Some large bequests to hospitals have been reported during the past week. Liverpool and Manchester institutions have again been fortunate. Mr. George Henry Ball, of Gambier Terrace, Liverpool, whose estate has been proved at over £200,000, left £2,000 to the Royal Southern Hospital, Liverpool, and £1,000 each to the David Lewis Northern Hospital, the Liverpool Royal Infirmary, and the Victoria Central Hospital, Liscard. The will of Mr. James Thomas Blair, of Whalley House, Whalley Range, Manchester, a leading shipping merchant in that city, has been sworn at £155,537. To the Manchester Royal Infirmary the testator bequeathed £2,000, and to St. Mary's Hospital, Manchester, and Ancoats Hospital, Manchester, £1,000 each. There are many other charitable legacies, and the income from the residue of the estate is to be applied by trustees in relieving the distress of poor persons, particularly the aged poor of Manchester and Salford.

### LARGE GIFTS TO BLIND CHARITIES.

ON the death of Mr. Leopold Salomon last year it was found that his beneficence had not been confined to his gift of Box Hill to the nation, for under his will he had directed that his residuary estate should be divided by his executors amongst unsectarian charities. He stipulated that one-fourth of such residue should be devoted to blind asylums, ophthalmic hospitals, and institutions for the blind. The trustees, who are Sir George Cave, J.P., Mr. A. W. Anson, and the Public Trustee, are now able to estimate the minimum value of the estate at £120,000, and have proceeded to allocate one-fourth of that sum to charities for the blind. The fortunate hospitals are the Western Ophthalmic (16 beds), £3,000, and the Royal Eye (42 beds); the Royal Westminster Ophthalmic (40 beds) and the Royal London Ophthalmic (138 beds), £1,000 each. The National Institute for the Blind (which includes St. Dunstan's Hostel) receives £10,000, and the Royal School for the Indigent Blind, £2,000; miscellaneous charities for the blind have been awarded sums from £250 to £1,000. A further announcement will shortly be made as to the allocation of the balance of £90,000 amongst the other charitable institutions, a number of which must be awaiting with lively interest the intentions of the trustees.

### THE TRAINING OF THE BLIND.

IT is a boast of Manchester that there is no city in the land which is ahead of it in regard to the treatment of the blind. The methods adopted, and the results achieved, at the great institution known as Henshaw's Blind Asylum—which Mr. Hayes Fisher, M.P., President of the Local Government Board, recently described as a model in every respect—are unique. Mr. D. H. Illingworth, the superintendent of Henshaw's, who was one of those who gave evidence before the Departmental Committee, declares that he sees no reason why every blind person of normal mentality should not be able to earn his or her living. The treatment adopted with such outstanding success at Henshaw's is based on the first principle of finding the blind something useful to do. Instruction in a remunerative employment follows, and in a short time they are able to earn their own livelihood. Wonderful results have been obtained at the Gresham Home, where thirty men are accommodated. Whatever work they can do is placed within their reach—for instance, such simple tasks as plaiting yarn, splitting canes, and drawing nails from packing cases. Within six months of the opening of the Gresham Home all the first batch of occupants had, of their own free will, gravitated into the working home, later being taught a trade in the ordinary way. It is understood that these methods of dealing with the blind are to be adopted under the State scheme.

### LECTURES ON TUBERCULOSIS.

THE Young Men's Christian Association has done so much for the welfare of our Army that we are not surprised to learn of one more instance in which they are endeavouring to help the soldier. The National Council of the Association has decided to undertake an educational campaign against tuberculosis throughout the Army, both in this country and abroad. Lectures are to be given in the huts erected by the Association. The Edinburgh Royal Victoria Tuberculosis Trust have presented a cinema film, "The Story of John McNeill," to illustrate the lectures. The first address will be given by Dr. Halliday Sutherland, consulting tuberculosis officer for North Marylebone, on Tuesday, September 4, at 7.30 P.M., at the Central Institute of the Young Men's Christian Association, Tottenham Court Road. The lecture will be on "Consumption, its Causes and Cure." The Rev. Archibald Fleming, D.D., of St. Columba's, will preside.

### PLAGUE AT GRAVESEND.

THE steamer *Matiana* arrived at Gravesend from Bombay on August 13. During the latter half of July, nine cases of plague occurred on board; six of these died, and were buried at sea. All the cases occurred amongst the lascar crew. Three convalescent cases were taken off the ship at Gravesend, and were removed to the Denton Hospital. The members of the crew were kept under observation, and since the arrival of the ship one further case has occurred, and the patient died in hospital. The *Matiana* had called at Falmouth two days before reaching Gravesend, but no passengers were landed and no cargo was discharged. The ship is still at the official mooring station off Gravesend, and the cargo is being discharged into lighters, the whole operation being conducted under the supervision of the Medical Officer of Health of the Port of London Sanitary Authority. The greatest care is being taken to prevent any spread of the disease, and all the circumstances of the case have been fully investigated by one of the medical inspectors of the Local Government Board.

### A HOSPITAL SHE-GOAT.

TEIGNMOUTH HOSPITAL was presented lately with a she-goat by Mr. Orsman, of Sheldon. At a sale at the hospital 1,277 tickets were sold for it in a raffle, from which the hospital received the sum of £40. The winner of the goat was the honorary secretary of the hospital, Mr. W. D. Oliver, who then returned the animal. What its future is to be we do not know, but it is a proud time for goats, which are everywhere fetching high prices. Some people who have given up poultry on account of the cost of poultry-food have taken to goats with great success. For the goat, which is easy to feed, has many uses. Apart from its milk, which in some districts is in demand owing to the shortage of cow's milk, the goat is coveted by the Ministry of Munitions, for it has the power of detecting the approach of gas before human senses are aware of it. Its use at the Front, therefore, for this purpose is an extension of the function it has already fulfilled in the mines, where warnings of gas are equally necessary. If the Teignmouth

Hospital wishes to dispose of its goat, it is an advantageous time to do so.

### THE SOLDIER AND THE CIGARETTES.

In the current number of the *Lancet* appears a report of a special research as to the cause of "soldier's heart." It was undertaken at the instance of the Medical Research Committee by Dr. John Parkinson and Mr. Hilmar Koefod. The report states that the investigators came to the conclusion that though excessive cigarette smoking is not the essential cause in most cases of "soldier's heart," yet it is an important contributory factor in the breathlessness and præcordial pain of many of them. Thirty smokers were taken, of whom twenty were cases of "soldier's heart" and ten were healthy men. Each subject smoked four or five cigarettes during a period of forty minutes. A definite effect was found in seventeen of the twenty patients, but it is interesting that the three who were not affected did not "inhale." Nine of the ten controls, all inhalers, were influenced, but not to the same degree. On the whole it may be said that in health the smoking of a single cigarette by an habitual smoker usually raises the pulse rate and blood pressure to a perceptible degree, and that these effects are more marked in patients suffering from "soldier's heart." It is clear that soldiers should be specially cautioned against "inhaling."

### POLITICIANS AND COTTAGE HOSPITALS.

We have become accustomed to the idea of a member of Parliament who does not need his salary handing over all or part of it to a hospital. But the commemoration of political events by gifts to hospitals is less common. A recent case concerns the Ashford Cottage Hospital, which lately received the sum of £1,000 from the Right Hon. L. Hardy, M.P., on his completion of twenty-five years as the representative of the Ashford or Saltburn division of Kent in the House of Commons. The gift is in War Loan Stock, and is being used to endow an "Evelyn Hardy" bed in memory of the donor's late wife. In Ashford, which is at once a busy junction and the centre of an agricultural district, the cottage hospital serves two different classes of the community. Its member has done something for both in helping the cottage hospital at Ashford. May the politicians follow his example in other constituencies!

### ASHTON DISTRICT INFIRMARY.

Intended primarily for the accommodation of wounded soldiers, the extensions at the Ashton District Infirmary have been opened by Mrs. John Summers, of Stalybridge. The new wards, which have cost £4,000—all of which has been subscribed —are to be known as the Summers Wing. With the exception of the infirmaries in the big cities, and the one at Wigan, the Ashton institution is now the largest of its kind in the County Palatine. Since the beginning of the war the infirmary's expenditure has risen from £7,000 to £10,000 per annum, and the adverse balance is now over £5,000. The President, Mr. Allen Shaw, expresses the opinion that there is little hope of reducing the expenditure in the future.

### KING EDWARD VII.'s HOSPITAL.

Mrs. Ivor Griffith, of Penhill, Cardiff, the wife of a shipowner who is serving with the Colours, has forwarded 1,000 guineas to King Edward VII.'s Hospital, Cardiff, for the endowment of a bed.

### PEMBROKESHIRE AND THE WELSH MEMORIAL.

The Pembrokeshire County Council has decided still to remain outside the Welsh National Memorial scheme. After a heated discussion at the Council the report of the Public Health Committee was accepted. This proposed that the Insurance Committee should be allowed to make its own arrangements, and that the County Council should allow the Public Health Committee to approach the Insurance Commissioners, or, if need arose, the Local Government Board for its assistance. Sir Evan Jones described the report as "a hopeless confusion of administrative bankruptcy," and indeed it is hard to say what it means. Surely, he added, Pembrokeshire did not want to be the one county to fail to deal with tuberculosis because of a "fancied oligarchy" at Cardiff. Sir Ivor Philipps, M.P., took the other side, and after Colonel Roberts had declared that the Association wished to capture the county because it was in dire need of funds through reckless expenditure, the Public Health Committee's report was adopted. Having adopted it, when will serious work begin?

### THE CHAPLAIN OF BRACEBRIDGE ASYLUM.

By eight votes to five the members of the Lincolnshire County Asylum Board, upon the recommendation of the House Committee, have appointed as chaplain of the Bracebridge Asylum the Rev. G. H. Trimble, at a salary of £125 per annum. Mr. Trimble is curate to the Rev. E. S. Smith, who has just resigned the chaplaincy, and for over six years has frequently officiated as Mr. Smith's deputy at the asylum. The appointment was opposed by the Mayor of Lincoln and others, on the grounds that the terms of the appointment should be reconsidered and that the vacancy ought to be advertised. The Clerk, on being appealed to, said that if the Board were appointing a fresh officer the vacancy would be advertised, and Alderman White, who seconded the amendment to discuss the matter in committee, contended that Mr. Trimble would be a fresh officer. The amendment, however, was lost.

### THE LINCOLNSHIRE COUNTY ASYLUM BOARD CEMETERY.

The desire of many Nonconformist patients to be buried in the consecrated portion of the asylum cemetery was an interesting fact which emerged in a discussion at the last meeting of the Lincolnshire County Asylum Board. The Home Office wrote stating that if the burial-ground was to be extended they had no objection to the consecration of a part only. The Clerk observed that the only difference between consecrated and unconsecrated ground was the question of burying Nonconformist patients. A Church of England clergyman might object to officiate at an interment in unconsecrated ground. A member said another question was "Could a Nonconformist be buried in consecrated ground?" Another member remarked that his experience was that nineteen out of twenty Nonconformists wanted to be buried in the consecrated portion, and a third said that at the recent meeting somebody made the remark that these people had had a hard life here, and if there were any benefit in being interred in consecrated ground by all means let them have that benefit. It was thereupon decided to consecrate the whole of the additional ground.

### A MATERNITY HOME FOR MORLEY.

Sir Charles Scarth, of Scarthingwell, Morley, has bought Morley Hall with its extensive grounds, formerly the residence of the late Mr. Oliver Scatcherd, and presented it to the borough, of which he has been six times Mayor and of which he is a freeman, for use as a maternity home and babies' welcome. This splendid gift on the part of one of the largest cloth manufacturers in the district has been received with great gratitude not only by the local authority, but also by that class in the community which it will chiefly benefit. The Hall is an ancient mansion, built in 1683, and is delightfully situated on the border of Scatcherd Park, which is public property.

### WESTMORLAND AND CHILD WELFARE.

A comprehensive maternity and child-welfare scheme, which is to apply to the whole of the county of Westmorland except the borough of Kendal, has been submitted to the local authorities concerned by the Westmorland County Council. The proposal embraces the utilisation of the staffs of the existing nursing associations, and the payment of 50 per cent. of the expenses incurred, the appointment of additional nurses in areas not already covered, such nurses to act also as health visitors, and the provision of maternity beds in institutions. The scheme may be amended in its details, but that it will be adopted so far as its main outlines go is a certainty.

### INFANT WELFARE RESULTS.

Whilst many local authorities are now beginning campaigns to prevent wastage of child life, Leicester is able to report results. For last year the infantile mortality was only 104.8 per 1,000 births, this being the lowest rate on record, and it indicates a very substantial reduction, which, as Dr. Millard, the medical officer of health, says in his annual report, is all the more satisfactory in that it coincides with the very special efforts made both by municipal and voluntary agencies to combat infantile mortality and preserve infant life. The work is bearing fruit. When it is considered that so many mothers are at work and away from home the greater part of the day, and that in consequence many more infants have had to be put out to nurse than was the case in pre-war times, this reduction in infantile mortality is all the more remarkable. There is, however, one consideration to be borne in mind. Owing to the increased number of marriages on the one hand, and the absence of husbands on the other, an unduly large proportion of the births now occurring must, it may be presumed, be the result of recent marriages. In other words, they are "first babies," and "first babies" usually come in for much more care and attention than those which arrive in families already "overrun" with children, and whose advent, in many cases, is not particularly desired. Still another consideration is that the mothers in these cases are often earning abnormally good wages, and, in the case of soldiers' wives, are also in receipt of the separation allowance, so that poverty—that arch-enemy of infant life and one of the most fruitful indirect causes of infantile mortality—has been comparatively rare. In this connection Dr. Millard recalls the investigation made some years ago by the medical officer of health for Birmingham, as the result of which he expressed the opinion that the advantage of the extra income earned where mothers went out to work tended to neutralise the undoubted disadvantage of putting the child out to nurse during the day.

### THE UNDER-PAYMENT OF DENTISTS' MECHANICS.

Dental surgeons, who have to complain of the difficulty in obtaining platinum out of which the pins in supernatural teeth are made and of the scarcity of mechanics, are not the only persons in their profession who suffer by the war. There is a reverse side to their grievance. This is the grievance of the mechanics themselves, who in many cases have been removed from the combatant ranks to take up dental work in the Army, on which they are employed without receiving the pay due to skilled work. The sympathy to which they are entitled can be seen when the case of jewellers' assistants is considered. The following case is noteworthy. A jeweller's assistant after enlistment was set to work on mending the sights of damaged guns at an English arsenal. After proving himself competent at making the delicate adjustments required, for which his previous experience provided a valuable training, he was able to earn as much as £3 or more a week. If, as is clear, he is entitled to this high pay for skilled work, those who are set to the actual skilled work for the Army on which they were engaged before enlistment are, to say the least, entitled to similar recognition. We hope the Army will not be deaf to so obvious an argument.

### CONSUMPTION IN LONDON.

The President of the Local Government Board, Mr. Hayes Fisher, recently received a deputation from the London Insurance Committee on the present unsatisfactory condition of those insured people in London who are suffering from phthisis. The members of the deputation pointed out that many of the insured persons in London were not receiving the benefit to which they were entitled, and towards which they had paid. Owing to the lack of funds the 932 beds which should have been provided were not provided, and only 400 beds were available. As a result of this there was a waiting list of over 300 people. In the year 1915 there was a deficiency in the Committee's funds of £6,000. It was suggested that the Local Government Board should bring pressure to bear on the London County Council so as to induce them to come into the scheme, and so render available £70,000 for the treatment of the tuberculous of London. Two-thirds of the patients applied too late. The great need for the successful treatment of tuberculosis is early sanatorium treatment. Many patients only come for treatment when it is too late for any real good to be done. If patients could not be admitted to a sanatorium as soon as they applied, the delay might easily be the cause of the loss of the patient's life. The President of the Local Government Board gave a sympathetic but indefinite answer.

### THE MINISTRY OF LABOUR AND THE ASYLUM WORKERS' UNION.

The refusal of the Lancashire Asylums Board to recognise the National Asylum Workers' Union, whose recent manifesto has been published in The Hospital, has had an important sequel. At a special meeting of the Board, presided over by Sir Norval W. Helme, a letter was read from Sir George Askwith asking if the Board was willing to refer the matter to arbitration. With this was received a copy of a letter addressed by the Union to the Ministry of Labour. Alderman Taggart, who said that the Union was even now considering a strike, moved that the Board inform the Minister of Labour that it would agree to arbitration. Alderman Shelmerdine asked how the Board could agree to arbitration with a Union which, so far as it was concerned, did not exist. Sir Harcourt Clare, the clerk, urged that it was a question of public policy which rested with the Government. By a narrow majority of two it was decided to agree to arbitration. It was left with Sir Harcourt Clare to ask the Ministry of Labour to accept the responsibility of dealing with the matter. The reasons for the refusal of the Board to recognise the Union are also being placed in Sir George Askwith's hands.

### DISPENSING WITH GLYCERINE AND SUGAR.

Means of dispensing with sugar and glycerine —or rather of dispensing without them—are suggested in a circular which has been issued to the medical profession by the Economy in the Use of Drugs Committee which was appointed by a Government Department at the beginning of the war. It was on the advice of this Committee, so it would appear, that the General Medical Council took the action, noted in The Hospital of August 11, of withdrawing from the British Pharmacopœia preparations containing sugar and glycerine, and the Committee's object in circularising doctors is to impress upon them the necessity, in the public interest, of refraining from prescribing compounds containing either of these substances save for those patients for whom they are imperative. A useful substitute for glycerine or sugar for improving the palatability of a mixture is chloroform water and mucilage of tragacanth, and the formula suggested is powdered gum tragacanth 30 grains, chloroform 40 minims, water to 10 ounces. Various "glycerine substitutes" have been placed on the market, but for practical purposes the above formula is quite suitable, while in many cases no harm would be done by refraining from prescribing either glycerine or sugar or any substitute. It is hoped that before long supplies of saccharin will be more plentiful, and then the difficulty of making bitter medicines sweet will be less acute, although, of course, saccharin has its limitations. The fact that the wholesale druggists are still in a position to supply sugar and glycerine preparations ought not to influence prescribers in their decision to prescribe medicines without these two substances.

### THIS WEEK'S DRUG MARKET.

Quiet conditions still prevail in the drug market so far as actual business is concerned, but there is a fair amount of inquiry, which may lead to orders in due course. Values are well maintained, but actual price fluctuations have not been numerous. Of cocaine there is almost an entire absence of offers, and for any small parcels available higher prices are asked. The advanced price of menthol is maintained, and Japanese dementholised peppermint oil is again rather dearer. Bismuth preparations are unchanged in price, and makers are able to meet the steady demand. In one of the home counties the lavender plant has been distilled, and the grower reports a poor yield; a few weeks ago the plant looked well, but the recent rains did no inconsiderable damage; in another district distillation has not yet begun. The English peppermint crop, which looks poor, will be gathered in about a fortnight. Essence of lemon has a downward tendency in price, and the demand is quiet. Caraway oil is much dearer. Oils of cloves and almonds are also dearer. The value of quinine still has an upward tendency, but buying is restricted to a small scale.

### TO OUR READERS.

**Contributions are specially invited from any of our readers to these columns. They should deal with topical subjects and news. They must be authenticated for the information of the Editor only. The minimum payment if published is 5s. There is no hard-and-fast rule as to space, but notes of about twenty lines in length are preferred.**

